# Supplementary material for: Attentional selection drives information convergence within macaque lateral prefrontal cortex
Source: PNAS Nexus. 2026 Sep 16;5(9):pgag311. doi: 10.1093/pnasnexus/pgag311 (PMC13618800; doi:10.1093/pnasnexus/pgag311)
Supplement: Supplementary file 1 [file pgag311_supplementary_data.docx]

**Supplementary material**

*Supplementary Table 1. Numbers of analysed channels and neurons across sessions in each area.*

| Animal | Area | Number of channels | | Number of neurons | |
| --- | --- | --- | --- | --- | --- |
|  |  | Median | Range | Median | Range |
| A | vLPFC | 17 | 9-31 | 21 | 10-55 |
|  | dLPFC | 16 | 8-33 | 21 | 10-63 |
| B | vLPFC | 8 | 5-23 | 9 | 8-29 |
|  | dLPFC | 9 | 6-24 | 10 | 7-29 |

*Supplementary Figure 1. Schematic overview of the analysis approach. HFP = high-frequency power; LDA = linear discriminant analysis; CV = cross-validation; TFCE = threshold-free cluster enhancement.*
